# Biomimetic Modification of Waterborne Polymer Coating Using Bio-Wax for Enhancing Controlled Release Performance of Nutrient

**DOI:** 10.3390/polym17070838

**Published:** 2025-03-21

**Authors:** Lianjie Wan, Cong Ge, Fei Ma, Jianmin Zhou, Changwen Du

**Affiliations:** 1 The State Key Laboratory of Soil and Sustainable Agriculture, Institute of Soil Science Chinese Academy of Sciences, Nanjing 211135, China; wanlianjie@issas.ac.cn (L.W.); gecong@issas.ac.cn (C.G.); fma@issaa.ac.cn (F.M.); jmzhou@issas.ac.cn (J.Z.); 2 College of Modern Advanced Agricultural Sciences, University of Chinese Academy of Sciences, Beijing 100049, China

**Keywords:** controlled release fertilizer, bio-wax, biomimetic modification, rice, maize

## Abstract

Waterborne polymer coated controlled release fertilizers (CRFs) are highly valued for their potential to enhance nitrogen use efficiency (NUE) and reduce fertilization labor costs. However, their application in crops with long growth periods, such as rice and maize, is limited by inadequate coating strength and suboptimal hydrophobicity. Inspired by the hydrophobic and anti-fouling structure of lotus leaf cuticles, this study biomimetically modified waterborne polyacrylate-coated urea (PACU) using natural bio-wax including rice bran wax (RBW), candelilla wax (CAW), bees wax (BW) and carnauba wax (CW), along with paraffin wax (PW) as a control. The modifications significantly extended nutrient release duration by 22 d compared to unmodified PACU, with CW providing the longest duration, followed by CAW, BW, RBW, and PW. Additionally, the modification of BW, CAW, and CW exhibited superior hydrophobicity and affinity to polyacrylate coatings, while the inferior hardness and toughness of PW compromised its controlled release performance. Field trials demonstrated that CW-modified CRFs effectively controlled nutrient release in rice and maize, resulting in a 7.2% increase in rice yield and a 37.9% increase in maize yield, as well as an 18.7% improvement in NUE compared to conventional fertilizers. These findings offered a novel approach for hydrophobic modification of waterborne polymer coatings, thereby enhancing the performance and applicability of waterborne polymer coated CRFs in long-season crops.

## 1. Introduction

Fertilizer application serves as a fundamental pillar of contemporary agricultural systems, delivering critical nutrients to crops while significantly contributing to yield enhancement [1,2]. The extensive adoption of chemical fertilizers has played a crucial role in addressing global food security challenges and propelling agricultural advancement [3,4]. However, the excessive utilization of nitrogen-based fertilizers frequently exceeds crop nutrient requirements, triggering a cascade of environmental consequences. These surplus nutrients subsequently migrate into aquatic ecosystems and the atmosphere through leaching, denitrification, and volatilization processes [5,6,7,8], leading to multiple ecological challenges including water eutrophication, amplified greenhouse gas emissions, soil degradation, and escalated agricultural operational costs [9,10,11]. In response to these pressing issues, controlled release fertilizers (CRFs) have emerged as an innovative solution, designed to optimize nutrient release kinetics. This technological advancement demonstrates significant potential for enhancing nutrient utilization efficiency, boosting agricultural productivity, and minimizing ecological contamination [12,13]. It is noteworthy that “nanofertilizers” possess similarities to CRFs, as both are designed to precisely regulate the amount of fertilizer utilized by crops, thereby decreasing resource waste and minimizing environmental damage [14].

As the predominant category of CRFs, coated fertilizers feature a core-shell architecture comprising solid fertilizer granules encapsulated within hydrophobic organic/inorganic coatings, typically applied via spray deposition and thermal curing processes [15]. This engineered barrier regulates nutrient release kinetics through controlled diffusion across the semipermeable membrane interface. Consequently, the coating material selection critically determines three key performance parameters: (1) fertilizer efficiency optimization, (2) environmental footprint reduction, and (3) temporal alignment between nutrient availability and plant uptake patterns [16,17,18,19].

Conventional organic polymer coatings—primarily resin-based or thermoplastic systems—dominate commercial applications owing to their tunable release profiles and environmental resilience [20,21]. Nevertheless, these petroleum-derived materials present significant sustainability challenges, including non-biodegradability, potential for persistent environmental contamination, and reliance on ecotoxic organic solvents during membrane fabrication [22,23,24,25]. Polyacrylate (PA), a prominent waterborne polymer, is widely recognized for its remarkable film-forming characteristics, excellent adhesion, and cost efficiency [26]. Notably, studies have demonstrated that fertilizers coated with PA did not adversely impact soil properties or the activity of soil microorganisms [27,28,29]. Its versatility even extends to medical applications, where it served as a potential drug carrier for targeted therapies [30,31]. Thus, PA coatings emerge as an eco-conscious alternative, employing aqueous-phase synthesis to achieve superior film-forming characteristics and process-friendly viscosity without hazardous solvents [32,33,34]. However, the inherent hydrophilicity of PA compromises its sustained-release performance through accelerated membrane permeability, particularly limiting its applicability for perennial crops or agricultural systems requiring extended nutrient delivery [35,36].

Biological waxes, encompassing both zoogenic and phytogenic derivatives, represent naturally occurring protective biomaterials that function as either organismal secretions or nutrient reservoirs [37,38,39]. Characterized by their inherent hydrophobicity, these waxes demonstrate dual functionality as semipermeable barriers against moisture infiltration and microbial colonization [40,41,42]. Their chemical architecture—comprising eco-compatible constituents like long-chain hydrocarbons, free fatty acids, wax esters, and triterpenoids—positions them as ideal candidates for engineering superhydrophobic surfaces, thereby offering innovative potential for polyacrylate-coated fertilizer modification [43,44,45]. Previous investigations have established that carnauba wax (CW), extracted from Copernicia prunifera foliar structures, significantly enhances the controlled release properties of polyacrylate-coated urea (PACU) [46]. Nevertheless, its agronomic efficacy under field conditions remains undemonstrated. Comparatively, paraffin wax (PW), a petroleum-derived non-biological counterpart, has been industrially adopted as an exterior coating to optimize nutrient release profiles in bio-based CRFs [47].

This study introduced a biomimetic strategy for enhancing PACU through systematic wax modification. Four biological waxes with different melting points 38–41 °C, i.e., carnauba wax (CW), rice bran wax (RBW), candelilla wax (CAW), and bees wax (BW), were used to modify the surface hydrophobicity of waterborne polyacrylate. Additionally, paraffin wax (PW)-modified coated fertilizer and unmodified PACU served as control, and the effects of nutrient controlled release were evaluated, which was further validated by field trials.

## 2. Materials and Methods

### 2.1. Materials

Carnauba wax (CW) and candelilla wax (CAW) in the form of flakes were purchased from Shanghai Macklin Biochemical Co., Ltd. (Shanghai, China). Rice bran wax (RBW) and bees wax (BW) in pellet form were obtained from Nanfeng Wax Industry Co., Ltd. (Dongguang, China). Paraffin wax (PW) and potassium persulfate (KPS, chemical grade) were supplied by Sinopharm Chemical Reagent Co., Ltd. (Shanghai, China). Methyl methacrylate (MMA, AR), n-butyl acrylate (BA, CP), methacrylic acid (MAA, CP), and urea (AR) were purchased from Nanjing Chemical Reagent Co., Ltd. (Nanjing, China). Sodium dodecylbenzene sulfonate (SDBS, AR) was sourced from Chengdu Kelong Chemical Reagent Co., Ltd. (Chengdu, China), and nonyl phenyl polyoxymethylene ether-10 (OP-10, CP) was obtained from Hebei Xingtai Kewang Auxiliary Agent Co., Ltd. (Xingtai, China). The urea granules (46.6 wt.% nitrogen, 2.00–4.75 mm in diameter) were provided by Shandong Luxi Fertilizer Co., Ltd. (Liaocheng, China). Deionized water (pH 6.92, resistance 18.3 MΩ cm^−1^) was used throughout this experiment.

### 2.2. Synthesis of Polyacrylate Emulsion

The polyacrylate emulsion was synthesized by a semi-continuous emulsion polymerization method [48,49]. First, the aqueous phase was prepared by dissolving 8.24 g of OP-10 and 4.12 g of SDBS in 248 g of water in a three-neck flask. Simultaneously, an oil phase was formed by mixing 110 g of BA, 90 g of MMA, and 5 g of MAA. The oil phase was added to the aqueous phase and stirred vigorously for 30 min. Then, 75% of the resulting emulsion was removed from the flask, while the remaining portion was heated to 85 °C. An initiator solution (50 mL, 0.013 g mL^−1^ KPS) was prepared, along with the removed portion of the emulsion, divided into four parts. These portions were alternately added back into the reaction mixture, while maintaining a stirring rate of 200 rpm. After the emulsion and initiator were thoroughly added, the reaction was carried out at 85 °C for 3 h. Finally, the temperature was reduced to below 40 °C to complete the synthesis.

### 2.3. Preparation of Polyacrylate-Coated Urea (PACU)

Urea granules were coated in a Wurster fluidized-bed equipped with a bottom spray pneumatic nozzle (LDP-3, Changzhou Jiafa Granulation Drying Equipment Co., Ltd., Changzhou, China). The temperature was maintained at 45–50 °C, with a spray rate of 2.5 g min^−1^ and an atomization pressure of 0.1 MPa. After the coating process was completed, the coated urea granules were dried in an oven at 80 °C for 8 h. To prevent film adhesion between the urea granules, approximately 0.1% of talcum powder was added. Two different coating rates of polyacrylate-coated urea (PACU) were prepared, where the dry matter of the emulsion constituted 5% and 8% of the total weight of the urea granules, respectively.

### 2.4. Preparation of Wax-Modified Polyacrylate-Coated Urea (PACU)

The PACU was modified by wax (WPACU) in a rotary evaporator (RE-52, Shanghai Yarong Biochemical Instrument Factory, Shanghai, China). Accurately weighed amounts of PACU and various waxes were placed into a round-bottom flask. The flask was rotated to ensure thorough and even mixing of the PACU and wax, and then heated in a water bath. Following the heating process, the flask was cooled to room temperature while continuing to rotate, allowing the wax to be evenly loaded onto the surface of the PACU. Referring to previous studies and considering the effects of heating temperature, wax ratio added to PACU, and rotation speed on the wax loading [43], we investigated and optimized the loading conditions based on the properties of different waxes. PACU samples with wax loading amounts of 0.0%, 0.5%, and 1.0% were successfully produced.

### 2.5. Characterization of the Coating

Samples of PACU and WPACU, modified with different types of wax, were selected, cut, and mounted on supports. The surface and section morphology were scanned using a scanning electron microscope (JEOL, Peabody, MA, USA). The surface functional groups of WPACU were characterized by a handheld TruDefender Fourier transform infrared (FTIR) spectrometer (Trudefender FT, Thermo Scientific, Waltham, MA, USA). The coating film from WPACU was carefully removed and pressed onto the attenuated total reflectance (ATR) crystal. FTIR-ATR spectra of unmodified and modified coatings were obtained in the 4000–650 cm^−1^ range, with a spectral resolution of 4 cm^−1^. Each spectrum was recorded as an average of 64 successive scans. Background spectra were scanned prior to each sample to correct for atmospheric interference and instrumental noise. Pure waxes were characterized using the same procedure. Photoacoustic spectra of waxes combined with polyacrylates were recorded using a Fourier transform infrared spectrometer (Nicolet 6700, Thermo Scientific, Waltham, MA, USA) equipped with a photoacoustic cell (model 300, MTEC, Waltham, MA, USA). The samples were placed in a cell holding cup (diameter 10 mm, height 3 mm), and the cell was purged with dry helium (5 mL min^−1^) for 10 s to eliminate interference from CO_2_ and H_2_O. Scans were performed in the mid-infrared wavenumber range of 4000–500 cm^−1^ with a resolution of 4 cm^−1^ and 32 scans, using three different moving mirror velocities (0.16, 0.32, and 0.64 cm s^−1^) for depth profiling. Each sample was scanned three times at different locations to obtain an averaged spectrum. The average profiling depths of specific absorption bands under different modulation frequencies were calculated using the following function:

(1) $$\mu =\sqrt{\frac{D}{\pi \times f}}$$

where *μ* denotes the thermal diffusion length, *D* represents the thermal diffusivity, *D* ≈ 10^−4^ cm^2^ s^−1^ denotes the polymer materials, and 10^−3^ cm^2^ s^−1^ denotes the wax [50,51]. The modulation frequency *f* (Hz) is the product of the wavenumber and the moving mirror velocity. And the FTIR-ATR spectra were smoothed by wavelet transform and normalized prior to spectral analysis in MATLAB R2020b (The Math Works, Natick, MA, USA). The principal component analysis (PCA) was performed to elucidate the internal structure of the spectra. FTIR-ATR spectra under varying conditions were analyzed and processed using the “2D Correlation Spectroscopy Analysis” package in OrginPro 2024b (OriginLab Corporation, Northampton, MA, USA), resulting in the generation of synchronous and asynchronous two-dimensional correlation spectra.

### 2.6. Nutrient Release Profile

Accurately weighed 5 g of fertilizer (WPACU) was placed into a glass bottle containing 100 mL of deionized water. The samples were incubated at 25 °C (PPX-450B, Saifu Corporation, Shanghai, China), with three replicates for each treatment. The solutions were collected and replaced with 100 mL of deionized water on the 1st, 3rd, 5th, 7th, 9th, 12th, 15th, 18th, 23th, 28th, 33th, 38th, 43th, and 50th d, respectively. The urea concentration in the solution was measured using an FTIR spectrometer (Nicolet 6700, Thermo Scientific, Waltham, MA, USA) equipped with ZnSe crystals, following the method described by Shen et al. [52]. To prepare a calibration curve, a series of urea solutions with concentrations of 0, 0.5, 1, 5, 10, 20, 30, 40, and 50 g L^−1^ were prepared. The FTIR-ATR spectra of the solutions were obtained by averaging 32 successive scans at a moving mirror velocity of 0.32 cm·s^−1^. The spectra were then smoothed by wavelet transform in MATLAB R2020b. The peak area corresponding to the C=O stretching vibration at 1750–1340 cm^−1^ was used to build the calibration curve for urea concentration. The urea concentrations (c) in released solutions were determined using the calibration curve. The urea release was calculated using the following formula:

(2) $$R=\frac{c\times V\times 10^{-3}}{m\times \rho }\times 100\%\;$$

where *c* is the measured urea concentration in solution (g L^−1^), *V* is the volume of the released solution (mL), *m* is the weight of coated urea (g), and *ρ* is the urea content in the modified coated urea (%).

### 2.7. Field Experiments

The rice field trials were conducted at Xinji Town, Yizheng City, Yangzhou City, Jiangsu Province, China (119°17′21.5″ E, 32°33′24.5″ N). The soil types in the region’s farmland are loam (Mollisols), and the basic agrochemical properties of the soil were as follows: pH 7.23, organic matter content 35.3 g kg^−1^, total nitrogen 2.0 g kg^−1^, available phosphorus 27.0 mg kg^−1^, and available potassium 102.0 mg kg^−1^.

The rice variety selected was “Hao Liangyou 1209”. The experimental design included four treatment groups: (1) no nitrogen fertilizer (CK), (2) conventional fertilization (CF), (3) carnauba wax-modified PACU (N1), (4) carnauba wax-modified PACU, reducing urea by 20% (N2). The fertilizer dosage was shown in Table 1. The experimental plots covered an area of 20 m^2^ (4 m × 5 m), with each treatment replicated three times. The plots were arranged in a randomized design, with a single-row configuration and protective rows around each plot. All management practices, except for fertilization, were consistent across treatments. On 25 June 2024, the plots were prepared by dividing them into treatment zones, constructing ridges, applying base fertilizers, and transplanting 15-day-old seedlings with 4 leaves. Four seedlings were planted per hole. Tillering fertilizer was applied on 15 July, followed by fertilizers for the jointing–booting stages on 8 August.

The CW-modified coated urea (40% N) used in the field experiments was supplied by Jiangsu ISSAS New Fertilizer Engineering & Technology Co., Ltd. (Yizheng, China). And the coating of the carnauba wax-modified polyacrylate-coated urea was 8%, with the carnauba wax load set at 0.5%. Additional fertilizers used in the experiments include ordinary urea (46% N) from Shanxi Tianze Coal Chemical Group Co., Ltd. (Jincheng, China), potassium chloride (60% K_2_O) from Yantai Qifeng Chemical Co., Ltd. (Yantai, China), and granular phosphate fertilizer (12.0% P_2_O_5_) from Jiangsu Meile Fertilizer Co., Ltd. (Danyang, China). Rice seed samples were collected at maturity on 17 October. At rice maturity, three 1 m × 1 m samples were randomly selected from each plot to measure rice yield-related parameters, including the effective number of spike stalk, number of seeds, and 1000-grain weight. And the actual yield per plot was recorded after the grains were dried and threshed. The nitrogen content of rice in each sample was determined using the SmartChem 200 auto analyzer (AMS Alliance, Frepillon, France) after complete digestion with H_2_SO_4_-H_2_O_2_. To evaluate fertilization efficiency, the nitrogen use efficiency (NUE) was calculated using Equation (3) [53] as follows:

(3) $$\;NUE=\frac{N\;uptake\;in\;N\;treatment-N\;uptake\;for\;no\;N\;treatment}{Applied\;amount\;of\;N}\times 100\%\;$$

### 2.8. Statistical Analysis

One-way analysis of variance (ANOVA) was used to analyze the effects of various fertilization treatments on the number of spike stalk, seed number, thousand kernel weight, rice grain yield, nitrogen use efficiency of rice, hundred-seed weight, and yield of maize. Differences between treatments were determined by comparing their means using the least significant difference (LSD) at *p* < 0.05. All statistical analyses of the data were implemented in SPSS version 21.0 (Armonk, NY, USA: IBM Corp.).

## 3. Results

### 3.1. Variations and Morphologies of Wax-Modified Polyacrylate-Coated Urea (WPACU)

The five waxes investigated in this study exhibited distinct melting characteristics that dictated specific processing temperatures during wax-modified polyacrylate-coated urea (WPACU) preparation. As detailed in Table 2, BW and PW demonstrated melting transitions at approximately 65 °C, requiring a reaction temperature of 70 °C. For optimal processing efficiency, thermal settings were maintained 5 °C above the melting points of other waxes: 75 °C for CAW, 85 °C for RBW, and 87 °C for CW. These temperatures ensured rapid wax melting and efficient production.

The phase transition temperatures of waxes critically influenced both manufacturing processes and storage stability. Higher-melting-point waxes exhibited enhanced structural integrity during storage but required greater thermal energy input, whereas lower-melting-point variants demonstrated improved processability at the expense of temperature sensitivity during storage. All materials followed established coating deposition patterns where loading rates increased proportionally with dosage, while utilization efficiency demonstrated an inverse relationship [43].

Although rotation speed had a minimal effect on total coating thickness, it significantly impacted the uniformity of wax distribution on the fertilizer. To ensure an even wax coating on the fertilizer surface, it was crucial that the fertilizer particles moved irregularly while the wax was melted in the flask. According to previous researches [43], the equipment speed should exceed 80 r min^−1^ to prevent the fertilizer particles from settling at the bottom of the flask. However, for BW and PW, speeds above 110 r min^−1^ caused the particles to move quite rapidly, leading to adhesion to the flask wall. Our experimental results revealed that rotational speed (80–130 rpm) exerted minimal influence on total coating thickness but significantly affected wax distribution homogeneity. To achieve uniform surface coverage, irregular particle movement was essential during the molten phase. Notably, BW and PW processing required speed limitation to 110 rpm to prevent particle-wall adhesion, whereas RBW, CAW, and CW tolerated speeds up to 130 rpm due to their higher melt viscosities, resulting from lower phase transition temperatures.

In the optimized production process of wax-modified coating fertilizers, the heating temperatures are 70 °C for BW and PW, 75 °C for CAW, 85 °C for RBW, and 87 °C for CW. For the rotation speed and wax load rate, a rotation speed of 100 r min^−1^ and a 1% wax load are optimal from both performance and economic perspectives. Additionally, as shown in Table 2, the iodine values of CW and RBW were lower compared to the other two biological waxes, indicating that these waxes possessed greater chemical stability, making their coatings more resistant to chemical degradation. BW and CAW presented a more economical option compared to other bio-waxes.

**Figure 1:**
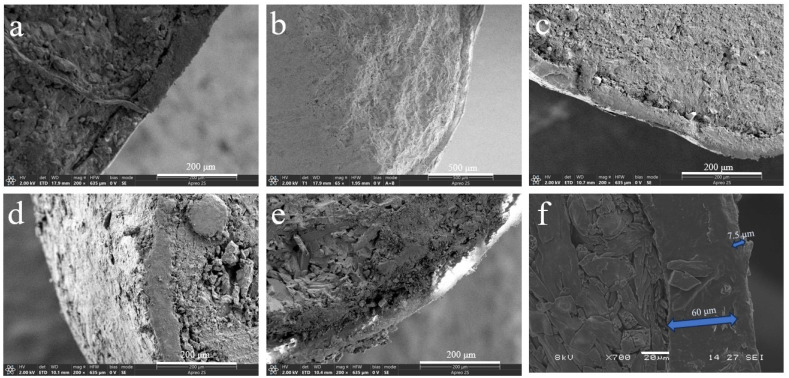
Scanning electron microscopy (SEM) images of sections (**a**–**f**) of coated urea. (**a**) The PACU with 8% content of PA. (**b**) The WPACU with 8% content of PA and 1% content of RBW. (**c**) The WPACU with 8% content of PA and 1% content of CAW. (**d**) The WPACU with 8% content of PA and 1% content of BW. (**e**) The WPACU with 8% content of PA and 1% content of PW. (**f**) The WPACU with 8% content of PA and 1% content of CW (The thickness of the WPA film was 60 μm, in contrast to the CW film’s 7.5μm).

The bio-wax-modified coated fertilizer comprises three primary components: a nutrient core with dual coating layers—polyacrylate (PACU) and bio-wax—both essential for achieving controlled nutrient release. Modifying PACU with different wax types significantly altered the morphology and internal architecture of wax-modified polyacrylate composites (WPACU), attributable to the unique physicochemical characteristics of each wax. As shown in Figure 1a, a well-defined interface exists between the fertilizer core and coating layers. Conversely, Figure 1b–f demonstrates effective interlayer fusion between polyacrylate and bio-wax coatings, evidenced by their blurred interfacial boundaries. This structural integration enhanced interlayer adhesion while minimizing coating delamination risks. The bio-wax modification effectively filled polyacrylate-derived surface voids caused by uneven coating application, thereby eliminating potential water infiltration pathways to the fertilizer core. Furthermore, bio-wax improved the consolidation of initially dispersed polyacrylate particles on the fertilizer surface, optimizing coating material utilization efficiency. Notably, Figure 1d,e reveals distinct delamination in BW and PW samples. Although partial wax detachment in cross-sections could originate from sample preparation artifacts, this phenomenon confirms the mechanical vulnerability of BW and PW coatings, particularly the low-hardness PW system. In contrast, CW, RBW, and CAW exhibited superior interlayer bonding with continuous transitional interfaces, demonstrating enhanced structural integrity for storage and transportation requirements.

### 3.2. Characterization of Wax-Modified Polyacrylate-Coated Urea (WPACU)

**Figure 2:**
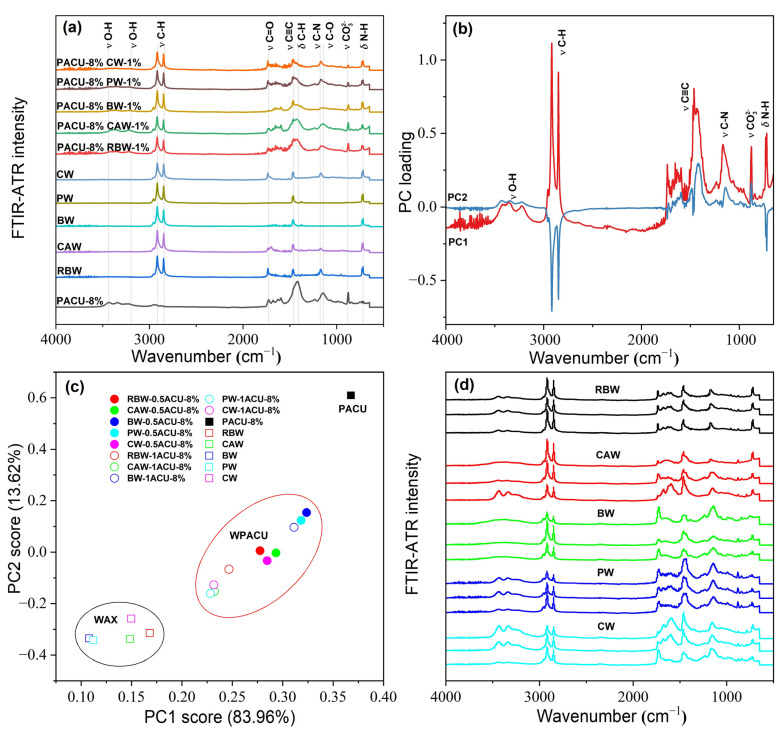
FTIR-ATR spectral characteristic of the wax and WPACU. (**a**) Comparison between the FTIR-ATR spectra of wax and WPACU. (**b**) Principal component loading of FTIR-ATR spectra for wax and WPACU. (**c**) Principal component scores of FTIR-ATR spectra for different PACU and WPACU. (**d**) Comparison among the FTIR-ATR spectra of the WPACU (8% PA and 1% wax) membrane at the end of the release phase.

The FTIR-ATR spectra of wax and WPACU were presented in Figure 2. Across the different waxes, similar characteristic peaks were observed at approximately 2915 cm^−1^, 1463 cm^−1^, and 720 cm^−1^, corresponding to C-H, C≡C, and N-H bonds [43,54], respectively. These peaks indicated the presence of aromatic aliphatic hydrocarbons and amides, contributing to the strong hydrophobicity of natural waxes. This hydrophobic nature enabled these waxes to effectively modify PACU and enhanced its water-repellent properties. The peak around 1174 cm^−1^ was attributed to the C–N stretching vibration from secondary amines, while the sharp peak at approximately 1730 cm^−1^ was associated with the stretching vibration of C=O from esters and diesters. Significant differences were observed between the five waxes, particularly for BW and PW, which exhibited weaker O-H and N-H peaks at wavenumbers above 3000 cm^−1^. This suggested a reduction in hydrophilic groups, indicating that BW and PW possessed stronger hydrophobic characteristics. The FTIR-ATR spectra of the WPACUs also exhibited peaks similar to those of the respective waxes, confirming the successful incorporation of waxes into the PACU coating and the transfer of hydrophobic properties to the polyacrylate film. Additionally, the presence of unique O-H peaks in the wax-modified membranes suggested that they retained a small portion of the hydrophilic characteristics of the original polyacrylate membranes. This balance allowed for the slow, controlled release of nutrients from the coated fertilizers without excessively prolonging the release period.

Among the five WPACUs, the variation in the O-H peak was most pronounced in the PACU modified with BW, indicating that BW showed the greatest impact on the polyacrylate film’s functional group composition. Furthermore, increasing the wax content reduced hydrophilic groups and enhanced the hydrophobicity of the coating, improving its controlled release properties. The FTIR-ATR spectra of WPACU at the end of the release process were shown in Figure 2d. Prior to urea release, the wax was evenly distributed on the surface of the polyacrylate film, and the spectra at different points were similar. However, after urea release, the characteristic peaks of the modified fertilizer film showed substantial differences compared to the pre-release spectra. This observation suggested that during the nutrient release process, the wax shedding or extension rate did not match the water absorption and swelling rate of the polyacrylate film. This mismatch could lead to uneven wax distribution on the polyacrylate film at certain stages, enabling water to enter the coating more rapidly, which accelerated the dissolution of the fertilizer core and enhanced the nutrient release rate.

Figure 2b,c shows the results of the principal component analysis (PCA) performed on the FTIR-ATR spectra. The first principal component (PC1) accounted for 83.96% of the total variance, highlighting that PC1 captured the majority of the variation in the FTIR-ATR spectra. The PCA score plot revealed three distinct clusters corresponding to wax, WPACU, and PACU. Both the wax and WPACU samples exhibited lower PC1 and PC2 scores compared to PACU, indicating differences in their spectral characteristics. The high loadings for PC1 and PC2 at the wavenumbers associated with hydrophobic functional groups (C–H, C=O, C≡C, and C–N) suggested that the modification with waxes enhanced the hydrophobicity of the polyacrylate-coated membrane.

**Figure 3:**
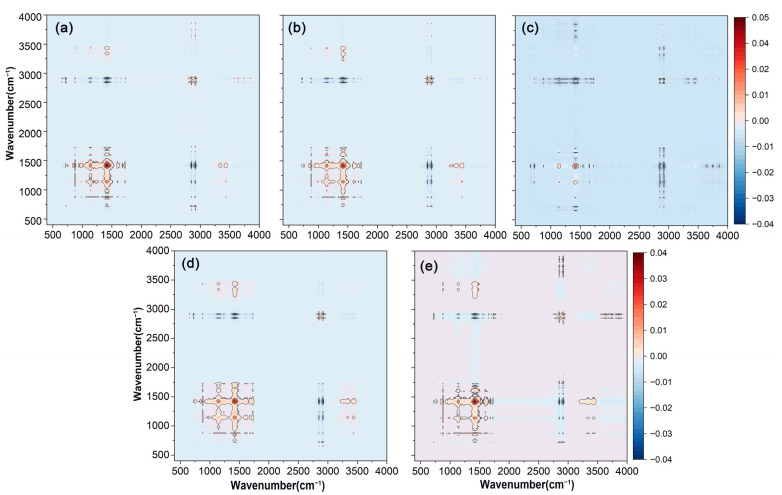
Two-dimensional synchronous correlation spectra were obtained from the FTIR-ATR analysis of polyacrylate-coated urea modified with varying amounts of wax. (**a**) RBW. (**b**) CAW. (**c**) BW. (**d**) PW. (**e**) CW. The variables in the FTIR-ATR spectra were PACU without wax, PACU with 0.5% wax, and PACU with 1% wax.

The two-dimensional synchronous correlation spectra of FTIR-ATR on the surface of various bio-wax-modified coated fertilizer films were presented in Figure 3. All five coatings exhibited similar automatic peak performance at approximately 2915 cm^−1^, 1463 cm^−1^, and 1174 cm^−1^, which was consistent with the results observed in one-dimensional spectra. The synchronous correlation spectrum provided insights into the simultaneous changes between two wavenumbers. Positive cross-peaks between C-O at 1174 cm^−1^ and C-H at 1463 cm^−1^ indicated that these functional groups responded similarly as the wax loading increased on the coating surface. In contrast, negative cross-peaks between C-O at 1174 cm^−1^ and C-H at 2915 cm^−1^ showed opposing sensitivities between these groups. Comparing the original data, it was observed that the spectral intensity at 2915 cm^−1^ increased, while the intensities at 1174 cm^−1^ and 1463 cm^−1^ relatively decreased, reflecting changes in the functional groups of the bio-wax.

**Figure 4:**
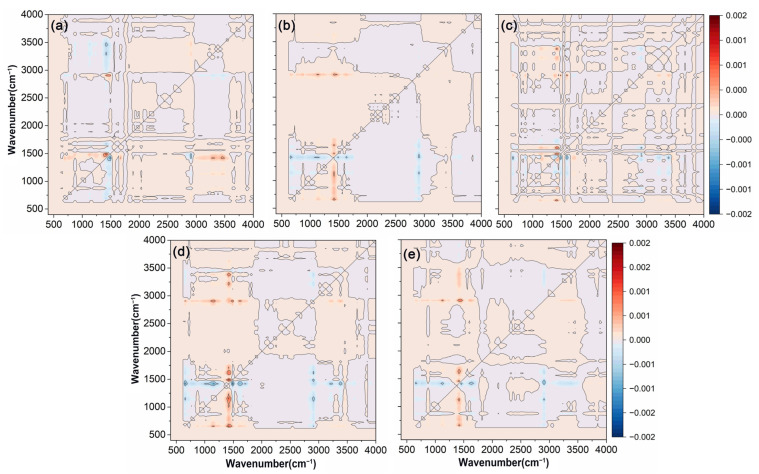
Two-dimensional asynchronous correlation spectra were obtained from the FTIR-ATR analysis of polyacrylate-coated urea modified with varying amounts of wax. (**a**) RBW. (**b**) CAW. (**c**) BW. (**d**) PW. (**e**) CW. The variables in the FTIR-ATR spectra were PACU without wax, PACU with 0.5% wax, and PACU with 1% wax.

Asynchronous correlation spectroscopy significantly enhanced the resolution of the original spectrum, enabling the detection of subtle spectral bands that were not easily identified in one-dimensional analysis. As depicted in Figure 4, cross-peaks between C=O at 1730 cm^−1^ and C-H at 1463 cm^−1^, as well as cross-peaks between C=O and O-H at 1463 cm^−1^ and 3200 cm^−1^, and 1463 cm^−1^ and 3444 cm^−1^, were discernible through asynchronous spectroscopy, which suggested that the functional groups corresponding to these wavelengths might be influenced by external factors, and their signal intensity changes occurred in a distinct sequence. Additionally, the presence of different spectral information at the two wavelengths corresponding to these cross-peaks suggested potential hydrogen bonding between the functional groups represented by these wavelengths [55]. Asynchronous correlation spectra not only improved spectral resolution, but also helped to determine the specific sequence of changes in different chemical groups under external disturbances [56]. The order of wavelength changes was 2915 → 1730 → 1463 → 1174, 3200, 3444 cm^−1^, with changes in the hydrophobic functional group C-H occurring earlier than those of other functional groups.

**Figure 5:**
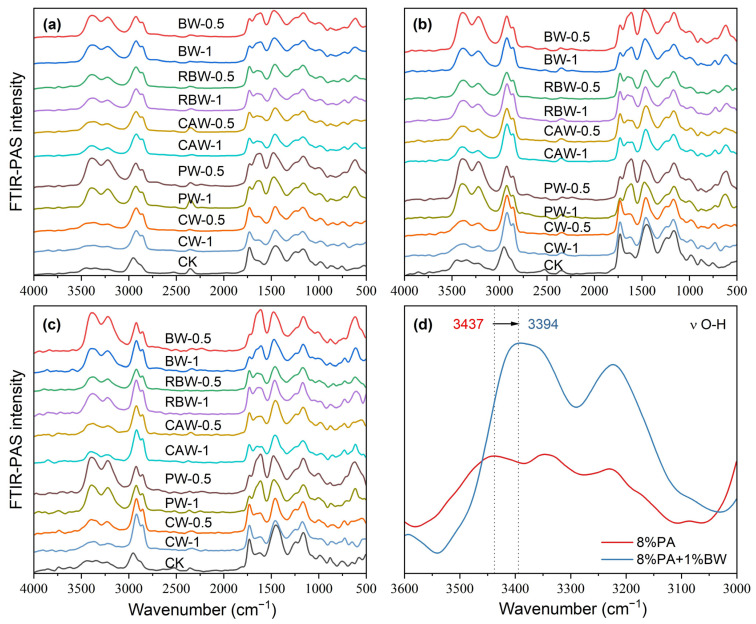
Photoacoustic spectra of different types of bio-wax coated fertilizers with different moving mirror rates. (**a**) The moving mirror rate is 0.16 cm s^−1^. (**b**) The moving mirror rate is 0.32 cm s^−1^. (**c**) The moving mirror rate is 0.64 cm s^−1^. (**d**) A redshift of the stretching vibration of O-H after BW modification.

Figure 5 presents the photoacoustic spectra of different types of bio-wax coated fertilizers. According to Equation (1), when the thermal diffusion coefficient is constant, the scanning depth depends on the moving mirror rate and the wavenumber. At moving mirror rates of 0.16 cm s^−1^, 0.32 cm s^−1^, and 0.64 cm s^−1^, the photoacoustic spectra reflected the group distribution within the membrane at different depths. At a moving mirror rate of 0.16 cm s^−1^, the detectable depth for wax at wavenumbers between 500 cm^−1^ and 4500 cm^−1^ ranged from approximately 6.6 µm to 19.9 µm. Since the film thickness was about 7.5 µm when the wax content was 1%, the photoacoustic spectrum at this rate primarily reflected the group distribution of the polyacrylate layer. Minor spectral fluctuations were likely due to density variations in the biological waxes, resulting in slight differences in coating thickness. At a moving mirror rate of 0.32 cm s^−1^, the detectable depth for wax at the same wavenumbers decreased from approximately 4.7 µm to 14.1 µm, while polyacrylates were detectable at depths from 1.5 µm to 4.5 µm. Since the detectable depth still exceeded the wax film thickness, the spectra for films with 0.5% and 1% wax content exhibited a similar group distribution. However, wax was still detectable at these depths, leading to more pronounced spectral fluctuations compared to uncoated wax films. At the highest moving mirror rate of 0.64 cm s^−1^, the detectable depth is further reduced from 3.3 µm to 10.0 µm. At this rate, the spectrum most accurately represented the interface between the wax and polyacrylate, providing the most relevant information on the interaction between these two layers.

Based on the distribution of functional groups, it appeared that the wax adhered to the polyacrylate through simple physical fusion. The most prominent peaks were observed at 3378 cm^−1^ and 2930 cm^−1^, corresponding to O-H and C-H groups, respectively. Across all types of wax, the wax coating showed a higher concentration of hydroxyl groups and fewer hydrocarbon bonds. To further elucidate the adhesion mechanism, the changes in peak positions between PACU-8% and BW-1%/PACU-8% were analyzed (Figure 5d). Notably, the O-H peak at 3437 cm^−1^ for PACU shifted to 3394 cm^−1^ for WPACU modified with BW. The red shift in the FTIR-ATR spectra suggested the formation of hydrogen bonds, which could reduce electron cloud density [57]. A similar phenomenon was observed for other wax-modified PACUs [43], indicating that hydrogen bonding might play a role in the adhesion between wax and polyacrylate.

### 3.3. Release Performances

**Figure 6:**
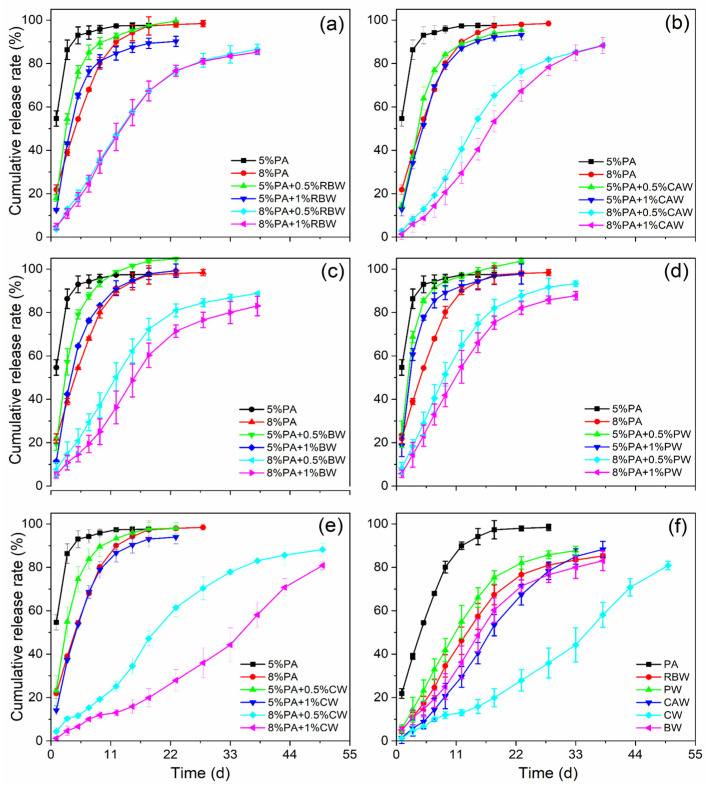
Release performance of wax-modified coated urea with different wax and PA contents. (**a**) RBW. (**b**) CAW. (**c**) BW. (**d**) PW. (**e**) CW. (**f**) Comparison of release longevity between WPACU with 8% content of PA and 1% content of wax, and three replicates for each treatment.

The controlled release profile demonstrated the effectiveness of the coatings for controlled release urea (CRU). The nutrient release profiles of different wax-modified polyacrylate-coated urea (WPACU) were presented in Figure 6. The addition of wax to the surface of the polyacrylate film significantly delayed nutrient release from the CRU, extending the release period by an average of 22 d compared to polyacrylate alone. As the wax content increased, the 24 h cumulative release rate of urea for PACU-5% and PACU-8% decreased, resulting in prolonged release longevity. In Figure 6a, increasing the RBW content did not notably extend the release longevity of PACU at the same polyacrylate content. Similarly, Figure 6b indicates that while the increasing CAW content smoothed the release curve, it did not significantly prolong release longevity. BW improved controlled release performance, with higher bees wax content extending the release duration and enhancing the release efficiency (Figure 6c).

As shown in Figure 6d, PW showed minimal impact on the release process. In contrast, CW (Figure 6e) demonstrated the most significant effect on extending release longevity and enhancing the release profile. Figure 6f shows the effect of PACU-8% modified with varying wax contents (1%). Compared to polyacrylate alone, the controlled release period of the five wax-modified fertilizers was extended by an average of 25 d, with CW exhibiting the longest extension, followed by CAW, BW, RBW, and PW. Additionally, the 24 h cumulative release rates for WPACU coated with RBW, CAW, BW, PW, and CW were 4.85%, 1.34%, 5.10%, 5.67%, and 1.16%, respectively. PACU modified with RBW, CW, and CAW exhibited lower 24 h release rates compared to BW and PW, likely due to the greater hardness of these waxes, which inhibited the initial swelling of the polyacrylate film. However, during later stages of release, the nutrient dissolution rate from PACU coated with RBW increased due to its poor toughness, causing the outer wax film to crack. In contrast, waxes with better toughness, such as CAW, CW, and BW, delayed this process, resulting in prolonged release times that better align with plant nutrient absorption. The unsatisfactory controlled release performance of PW could be attributed to its lower hardness and toughness.

**Figure 7:**
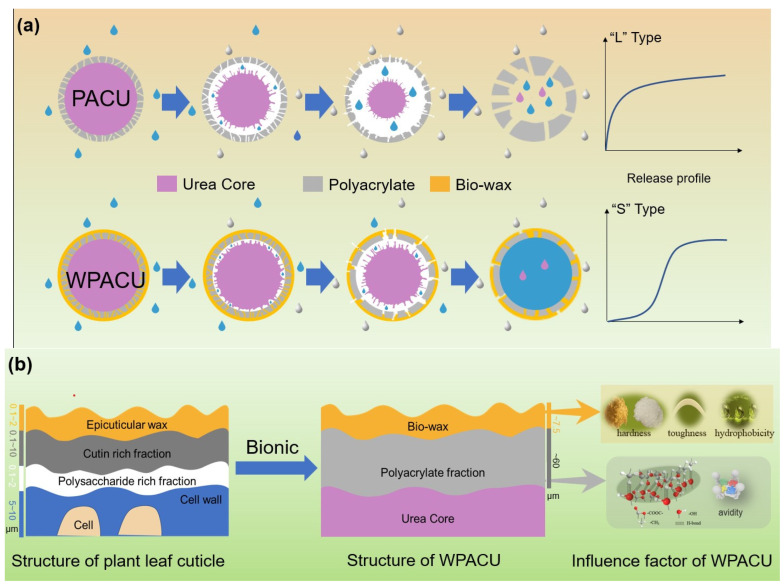
(**a**) Process of bio-wax biomimetic modification of PACU and film formation mechanism of WPACU. (**b**) The bio-wax-modified polyacrylate bionic double coating for controlled release fertilizer regarding the structure of plant leaf cuticle.

The plant cuticle, which covers the outer surface of epidermal cells, consists of two sublayers: the epicuticular wax layer and the hydrocarbon polymer layer (Figure 7) [58]. This cuticle layer effectively controls the movement of water and nutrients, ensuring optimal plant growth. Similarly, the objective of coated fertilizers was to regulate the transport of water and nutrients across the membrane, making the plant cuticle structure a useful model for fertilizer coatings. In this biomimetic approach, polyacrylate served as the hydrocarbon polymer layer (i.e., cutin, polysaccharides, etc.), while the bio-wax acted as the epicuticular wax layer. PACU alone showed insufficient hydrophobicity and was prone to water absorption and swelling, resulting in a shorter nutrient release lifespan. The incorporation of bio-wax into PACU improved the surface hydrophobicity of the coated fertilizer, preventing water ingress and extending the release duration. The physicochemical properties of different waxes and their affinity with polyacrylate influenced the controlled release performance of WPACUs to some extent. Additionally, PACU coatings tended to expand and crack due to their low thickness and insufficient strength (Figure 7a). When the polyacrylate coating rate increased to 8%, the initial nutrient release of the modified fertilizer was significantly reduced. At this stage, bio-wax not only blocked water penetration, but also slowed the swelling of the polyacrylate, thereby prolonging the “lag phase” of urea release. In the later stages, bio-wax limited membrane expansion, keeping it smaller than that of unmodified PACU, and prevented the significant enlargement of surface micropores, further extending the fertilizer’s release period.

According to the previous analysis, the primary factors influencing the properties and differences of five WPACUs were the hardness, toughness, and hydrophobicity of the wax, as well as its affinity with polyacrylate. Among the five types of waxes studied, CW exhibited the best controlled release performance. The interaction between the waterborne polyacrylate film, characterized by O-H and C=O bonds, and the carnauba wax layer, containing C-H and C=O bonds, was primarily mediated through hydrogen bonding. This hydrophobic architecture was beneficial as it impedes the penetration of water into the fertilizer core, thereby extending the urea release period. In the initial phase of release, the pronounced hardness and hydrophobic nature of carnauba wax not only deterred water ingress, but also decelerated the expansion of the polyacrylate coating, effectively elongating the “lag period” of urea release. As the process progresses, the exceptional toughness and hardness of carnauba wax ensured that the film’s swelling was more restrained compared to fertilizers coated exclusively with polyacrylate, leading to negligible surface expansion and a further protracted release of urea. Consequently, when juxtaposed with the other four waxes, carnauba wax’s superior hardness, toughness, and hydrophobic attributes significantly reinforced the coating’s durability and resistance during the nutrient release stages. These characteristics curtailed the swelling of the polyacrylate layer, reduced the nutrient release rate per unit time, and effectively extended the release duration. However, the high-melting-point (over 80 °C) posed processing challenges, a concern also noted for RBW and CW. In contrast, BW offered the advantage of easier processing, and their high toughness helped prevent breakage caused by the swelling of the polyacrylate coating, resulting in a more stable release rate during the middle and late stages of nutrient release. However, its lower hardness led to suboptimal performance during the early release phase. Although PW exhibited the poorest controlled release performance among the five WPACUs, its low melting point offered application potential due to reduced processing costs. Fertilizers coated with CAW exhibited a unique fluff-like surface morphology that significantly enhanced hydrophobicity and extended the release period.

Furthermore, CAW and BW were more cost-effective and had a longer release period than RBW, though their release durations were slightly shorter than CW, while being more affordable. Different bio-waxes showed considerable variation in controlled release performance, attributed to their distinct physical properties. Bio-waxes with higher hardness effectively limited the initial expansion of the coating and reduced early nutrient dissolution. Meanwhile, bio-waxes with greater toughness and affinity facilitated more efficient release during the middle phase, thereby extending the overall nutrient release duration. Overall, bio-waxes greatly improved the controlled release behaviors of waterborne polyacrylate-coated fertilizers, offering promising potential in nutrient controlled release.

### 3.4. Field Applications of Wax-Modified Polyacrylate-Coated Urea (WPACU)

The primary goal of modifying and developing coated fertilizers is to improve crop yield and quality. The yield results for rice were presented in Table 3. The use of carnauba wax-modified coated fertilizer (N1) led to a 7.2% significance increase in rice yield compared to conventional fertilizers (CFs), while the yield in the urea reduction treatment (N2) showed a slight decrease, though this difference was not statistically significant. N1 also exhibited a 18.7% higher nitrogen use efficiency (NUE) compared to CF, whereas the NUE of N2 did not differ significantly from CF.

Additionally, the seed number of N1 was significantly increased relative to CF, while no significant differences were observed in spike stalk number and 1000-grain weight. These results indicate that bio-wax-modified coated fertilizers could increase rice growth and yield. Furthermore, when the bio-wax-modified coated fertilizer was applied and urea input was reduced by 20%, no significant differences in either yield or NUE were observed compared to CF. These findings align with those of previous studies [59,60]. It may be attributed to the stable, slow-release nutrients from the bio-wax-modified coated fertilizer, which could reduce nutrient loss and supply the necessary nitrogen during rice’s critical growth stages.

Moreover, fertilizer trials were also carried out on maize, shown Table 4. For maize, the application of the waterborne polyacrylate-coated fertilizer (M2) resulted in a moderate increase in both hundred-seed weight and yield compared to the conventional treatment (M1). However, this improvement was not statistically significant. In contrast, the use of carnauba wax-modified coated fertilizer (M3) significantly increased both hundred-seed weight and yield compared to M1, with a notable yield improvement by 37.9%. The nitrogen content in maize treated with M3 was significantly higher than that with M1 and slightly increased compared to M2. According to Zhang et al., the bio-based coated fertilizer showed similar efficacy in enhancing corn production [61]. The future application of WPACU to maize could be combined with diverse farmland management strategies to fully leverage the benefits of controlled release fertilizers. These field trials confirmed that the carnauba wax-coated fertilizer showed a positive impact on crop yield, particularly for maize. However, the field trials of other bio-wax-modified coated fertilizers require further investigation. Especially for CAW and BW, which offer low cost, ease of processing, and good controlled release performance, their overall characteristics make them promising candidates for future modified wax coating fertilizers.

## 4. Conclusions

In summary, five types of wax-modified waterborne polyacrylate-coated fertilizers were developed. Bio-wax, with its superior hydrophobicity, hardness, toughness, and strong affinity for polyacrylates, provided a notable improvement in controlled release performance over paraffin wax and conventional waterborne polymer materials. Among the four bio-wax-modified fertilizers tested, the CW-modified coated fertilizer displayed the longest controlled release period, followed by CAW, BW, and RBW. The application of CW-modified coated fertilizers to rice and maize resulted in higher yields compared to traditional chemical fertilizers, with the notable and substantial increase observed in rice and maize. Thus, bio-wax serves as a natural, sustainable material that efficiently enhanced the slow-release performance of coated fertilizers, advancing the development of slow-release fertilizer technology in agricultural practices.

## Figures and Tables

**Table 1:** The fertilization treatments of rice.

Treatments	Base Fertilizer (kg ha^−1^)	Tillering Fertilizer (kg ha^−1^)	Jointing–Booting Fertilizer (kg ha^−1^)	Fertilizer (kg ha^−1^)		
				N	P_2_O_5_	K_2_O
CK	Phosphate fertilizer (750 kg), potassium fertilizer (90 kg)	0	Potassium fertilizer (60 kg)	0	90	90
CF	Phosphate fertilizer (750 kg), potassium fertilizer (90 kg) and urea (211.5 kg)	Urea (127.5 kg)	Urea (84 kg) and potassium fertilizer (60 kg)	195	90	90
N1	Phosphate fertilizer (750 kg), potassium fertilizer (90 kg), urea (148.5 kg) and CW-modified coated fertilizer (219 kg)	0	Urea (84 kg) and potassium fertilizer (60 kg)	195	90	90
N2	Phosphate fertilizer (750 kg), potassium fertilizer (90 kg), urea (81 kg) and CW-modified coated fertilizer (219 kg)	0	Urea (67.5 kg) and potassium fertilizer (60 kg)	156	90	90

**Table 2:** The partial indicators of waxes.

Wax	Source	Melting Point (°C)	Relative Density (g cm^−3^)	Iodine Value (mg (I_2_) g^−1^)	Price ($ kg^−1^)
RBW	Extraction and refining of rice bran oil.	78~80	0.970~0.972	7~10	35~60
CAW	Extracted from the epidermis of a candelabra shrub.	68~72	0.988~0.990	15~36	31~50
BW	Bees wax secreted from their abdomens.	61~65	0.954~0.964	8~23	12~30
CW	Extracted from the leaves and buds of the Brazilian palm.	81~86	0.996~0.998	5~14	50~102
PW	Extracted from petroleum, shale oil.	58~62	0.880~0.915	0~10	9~20

Note: The wax information was sourced from websites such as “ChemSrc” and “Chemical Book”.

**Table 3:** Effects of different treatments on the yield of rice.

Treatment	Number of Spike Stalk (10^4^ ha^−1^)	Seed Number	Thousand Kernel Weight (g)	Rice Grain Yield (kg ha^−1^)	Nitrogen Use Efficiency (%)
CK	253.4 b	99.3 c	22.7 a	5280.2 c	-
CF	334.5 a	114.8 b	23.0 a	8037.3 b	37.5 b
N1	331.6 a	121.3 a	23.3 a	8619.6 a	44.5 a
N2	346.1 a	109.6 b	22.9 a	7931.0 b	39.0 b

Note: CK, control; CF, conventional fertilizer; N1, carnauba wax-modified coated fertilizer treatment with equal nitrogen input; N2, carnauba wax-modified coated fertilizer treatment with 15% reduced nitrogen input. Values followed by different small letters in the same column indicate a significant difference among treatments (*p* < 0.05).

**Table 4:** Effects of different treatments on the yield of maize.

Treatments	Hundred-Seed Weight (g)	Yields (kg ha^−1^)
M1	28.01 b	6813.0 b
M2	30.98 ab	8196.1 ab
M3	35.16 a	9397.5 a

Note: M1, conventional fertilizer with multi-applications for maize; M2, PACU with an application for maize; M3, CW-modified PACU with an application for maize. Values followed by different small letters in the same column indicate a significant difference among treatments (*p* < 0.05).

## Data Availability

The original contributions presented in this study are included in the article. Further inquiries can be directed to the corresponding author.

## Abbreviations

The following abbreviations are used in this manuscript:

CAW	Candelilla Wax
CRFs	Controlled Release Fertilizers
CW	Carnauba Wax
BW	Bees wax
FTIR-ATR	Fourier Transform Infrared Attenuated Total Reflectance Spectroscopy
NUE	Nitrogen Use Efficiency
PACU	Polyacrylate-Coated Urea
PW	Paraffin Wax
RBW	Rice Bran Wax
SEM	Scanning Electron Microscopy
WPACU	Wax-Modified Polyacrylate-Coated Urea
